# Identifying concerns and needs in AYA survivors of pediatric cancer: a scoping review

**DOI:** 10.3389/fpsyg.2025.1669872

**Published:** 2025-12-04

**Authors:** Maria Jesus Moura, Margarida Custódio dos Santos, Luísa Barros

**Affiliations:** 1 Pediatric Psycho-oncology, Portuguese Institute of Oncology (IPO), Lisbon, Portugal; 2 CICPSI, Faculty of Psychology, University of Lisbon, Lisbon, Portugal; 3 Lisbon School of Health Technology – Polytechnic Institute, Lisbon, Portugal

**Keywords:** pediatric cancer survivors, childhood cancer survivors, difficulties, concerns, worries, needs

## Abstract

**Background:**

Adolescent and young adult survivors of pediatric cancer (AYA-CCS) require specialized, age-appropriate care throughout their lives. This scoping review was conducted in accordance with the Preferred Reporting Items for Systematic Reviews and Meta-Analyses Extension for Scoping Reviews (PRISMA-ScR) to identify and map self-reported difficulties, concerns, and needs among AYA-CCS.

**Methods:**

A comprehensive search was conducted in PubMed, Scopus, Web of Science, and EBSCOhost (PsycINFO and CINAHL) for articles published between 2014 and 2024. Eligible studies included first-person data from AYA-CCS (aged 15–39 years), addressing their cancer-related difficulties, concerns, or needs. Studies using qualitative, quantitative, or mixed methods were considered. Two reviewers independently screened studies and resolved discrepancies through discussion.

**Results:**

We screened 1,247 records, of which 24 studies met the inclusion criteria. The findings were classified into two main dimensions: (1) Concerns, including psychological burden, long-term effects, infertility and its impact on relationships, transition of care, fear of recurrence, and social, professional, and financial challenges; and (2) Needs, including tailored information, psychological support, and communication challenges.

**Conclusion:**

This scoping review highlights the multiple challenges faced by AYA-CCS, with emphasis on psychological burden. Survivors report needs related to information, psychological support, and communication. Flexible, age-adapted psychoeducational approaches may be beneficial. Involving survivors in program design could enhance relevance. Stratified research by age or developmental stage is essential to align care with evolving needs. A lack of standardized AYA-CCS definitions was identified, underscoring the need for uniform criteria to strengthen future research and care.

## Introduction

A pediatric cancer diagnosis can negatively influence the psychosocial well-being of patients, over the long term, and result in late effects during young adulthood and adulthood (Fardell et al., 2021). The majority of adolescents and young adult (ages 15–39 years, Janssen et al., 2021) childhood cancer survivors (AYA-CCS) who have received a cancer diagnosis in their childhood and are no longer receiving treatment (Bhatia et al., 2023), report at least one physical, emotional, or behavioral concern, with negative implications on their well-being (Plotka et al., 2021).

Throughout their development, they may experience psychosocial difficulties in schooling, professional life, financial stability, as well as in peer, intimate, and marital relationships (Warner et al., 2016; Frederiksen et al., 2019). As the survival rate of pediatric cancer patients increases, there is a growing focus on addressing their unique needs throughout their life course (Robison and Hudson, 2014), as well as their difficulties and concerns related to emotional distress and maintaining adherence to ongoing medical follow-up and survivorship surveillance (McDonnell et al., 2015).

The Dublin Declaration (Childhood Cancer International, 2016), prepared by the Childhood Cancer International (CCI) Survivors’ Network, pleads for professionals and stakeholders to acknowledge the unique challenges and needs of CCS as the foundation for their care. It calls for structured long-term follow-up and for survivors to be actively involved in decisions concerning their care. Along with this declaration, several studies highlight the need to make available interventions tailored to the individualized healthcare needs of this population (Bradford and Chan, 2017; Zhang et al., 2021; Osmani et al., 2021).

These multidisciplinary interventions should include some form of psychoeducational and psychological support (Kazak and Noll, 2015; Ryan et al., 2018; Phiri et al., 2023). Evidence from earlier collaborative cross-cultural research indicates that AYA-CCS experience specific unmet healthcare needs (Keegan et al., 2012), including a desire for support group participation, which underscores the need for targeted intervention programs.

Although several programs and studies have been developed to address the specific needs of AYAs through health promotion, psychological and psychosocial interventions (Bradford and Chan, 2017; Arpaci and Altay, 2024), few initiatives have specifically focused on adolescent and young adult childhood cancer survivors (AYA-CCS). Existing evidence indicates that the distinct difficulties and concerns of this population remain insufficiently examined, and only one systematic review to date has addressed the needs of AYA cancer survivors, without exploring their specific concerns or challenges (Galán et al., 2018).

Results consistently point to cancer survivors’ needs for individualized information and guidance, counseling, and psychological as well as social support. While several studies have documented the challenges faced by AYA-CCS after treatment and highlighted their primary concerns (Plotka et al., 2021; Janin et al., 2018; Jones et al., 2020), they have not explicitly examined the specific and multifaceted needs of this population.

While previous reviews have explored the psychological and social needs of adolescents and young adults (AYA) after cancer treatment (Keegan et al., 2012), only one systematic review to date has examined this population’s needs (Galán et al., 2018), without addressing the distinct difficulties and concerns of adolescent and young adult survivors of pediatric cancer (AYA-CCS). In line with the Dublin Declaration’s call to strengthen survivorship research and care (Childhood Cancer International, 2016), the present review advances the field by systematically synthesizing evidence on the psychosocial and survivorship experiences reported by AYA-CCS themselves. The insights derived from this synthesis are pivotal for guiding the development of tailored interventions that address the unique difficulties and concerns of this population, ultimately informing the design of more personalized and effective psychological support programs specifically for AYA-CCS.

To ensure an updated and comprehensive understanding of these survivors’ challenges, we formulated the following question that reflects the growing focus on this subject: What are AYA-CCS self-reported cancer-related difficulties, concerns, and needs?

The purpose of this study is to address the gap identified in the literature by mapping and synthesizing the available evidence on the self-perceived experiences of AYA-CCS. Specifically, it aims to identify and categorize the reported difficulties, concerns, and needs of adolescent and young adult survivors of pediatric cancer. This review seeks to provide a structured overview of the existing knowledge base, offering insights that may inform future research and guide the development of psychosocial support strategies tailored to this population.

## Materials and methods

A scoping review was performed using the framework: Preferred Reporting Items for Systematic Reviews and Meta-Analyses Extension for Scoping Reviews (PRISMA-ScR) (Tricco et al., 2018; Peters et al., 2020; Munn et al., 2018); the PRISMA-ScR checklist is provided as Supplementary File 1.

### Search strategy

The search strategy was guided by the PEO framework (Population, Exposure, Outcome) (Moola et al., 2015). PubMed, Scopus, Web of Science, and EBSCOhost (PsycINFO, CINAHL) were searched for English-language studies published between January 2014 and December 2024. Search terms were defined according to the PEO structure and are detailed in Table 1. Strategies were developed iteratively with an information specialist, combining controlled vocabulary (e.g., MeSH), free-text terms, Boolean operators (AND, OR), truncation (*), and wildcards to maximize sensitivity and precision. Reference lists of included studies were also screened.

**Table 1:** Search terms used according to the PEO framework.

PEO Component	Description	Keywords / Controlled Terms
Population	Pediatric cancer survivors	child* OR adolescent* OR “young adult*” OR “pediatric cancer survivor*” OR “childhood cancer survivor*” OR AYA
Exposure	Cancer survivorship	cancer OR “malignant neoplasm*”
Outcome	Difficulties, Concerns/worries, and Needs (These concepts are defined based on the APA Dictionary of Psychology) Difficulties: challenges or obstacles experienced by individuals, such as physical, emotional, social, cognitive, or financial hardships. Concerns/Worries: state of mental distress or agitation due to concern about an impending or anticipated event, threat, or danger. Needs: a condition of tension in an organism resulting from deprivation of something required for survival, well-being, or personal fulfilment	difficult* OR concern* OR worr* OR need*

Although conceptually aligned with the APA definition of ‘difficulties’, terms such as “challenge” and “obstacle” were excluded from the final search strategy, following pilot testing, as they lacked specificity and retrieved broadly framed or irrelevant records. Their exclusion was therefore a strategic decision to increase the precision of the search while maintaining conceptual alignment.

### Inclusion and exclusion criteria

Studies were included if they met the following criteria: (1) original empirical research (qualitative, quantitative, or mixed methods); (2) published in peer-reviewed journals in English between January 2014 and December 2024, a timeframe consistent with scoping review guidance to capture evolving literature (Munn et al., 2018); (3) included samples in which at least 50% of participants were adolescent and young adult (AYA) childhood cancer survivors (CCS), currently aged 15 to 39 years, who had completed curative treatment and were in full remission (4) reported first-person perspectives on difficulties, concerns, or needs, whether explicit or emergent.

Studies were excluded if they: (1) did not reflect survivors’ perspectives (e.g., reported only by caregivers or health professionals); (2) did not allow disaggregation of AYA-CCS data; (3) included only survivors of central nervous system tumors due to specific neurocognitive sequelae; or (4) were non-empirical publications, including reviews, editorials, and commentaries.

### Quality assessment of articles

The quality assessment of eligible articles was done using the Kmet et al., checklist (Kmet, 2004). This tool includes items to access the quality of both quantitative and qualitative studies with two checklist studies. The maximum score for qualitative studies is 20 and for quantitative studies 28. However, given the type of quantitative studies eligible for extraction, three items on this checklist were not applicable because these items assess experimental or interventional quantitative studies, so the maximum score was 22. For mixed studies, we merged both checklists, with 17 items and a maximum score of 34. Two reviewers (MJM and MLB) independently assessed the quality of the articles. Disagreements were solved by discussion. This multi-step process ensured methodological triangulation and consistency in study selection and data extraction.

### Data extraction and analysis

The research results were gathered in Rayyan^1^ (Ouzzani et al., 2016), allowing for different reviewers to analyze articles simultaneously. Initially, duplicates were removed. Then, the title and abstract of each study were analyzed for inclusion/exclusion decisions based on the defined criteria, independently by the two first authors (MJM e MCS). Afterward, these two authors analyzed the full texts to reach the final selection. Any disagreements between the first two authors regarding eligibility were solved by a third senior author (MLB). Then, the first author (MJM) collected data from the included studies, and the second author (MCS) reviewed the data for accuracy. Data was extracted and the following information summarized for each study: (1) study reference (author, year of publication, country where the study was conducted); (2) study objectives; (3) study design (quantitative, qualitative, or mixed methods); (4) participants (sample size, age range, years after treatment); (5) measurements (interviews, questionnaires, or surveys used to collect data); (6) main results (reported survivors’ perceptions of the difficulties, concerns and service needs); (7) quality score.

## Results

**Figure 1:**
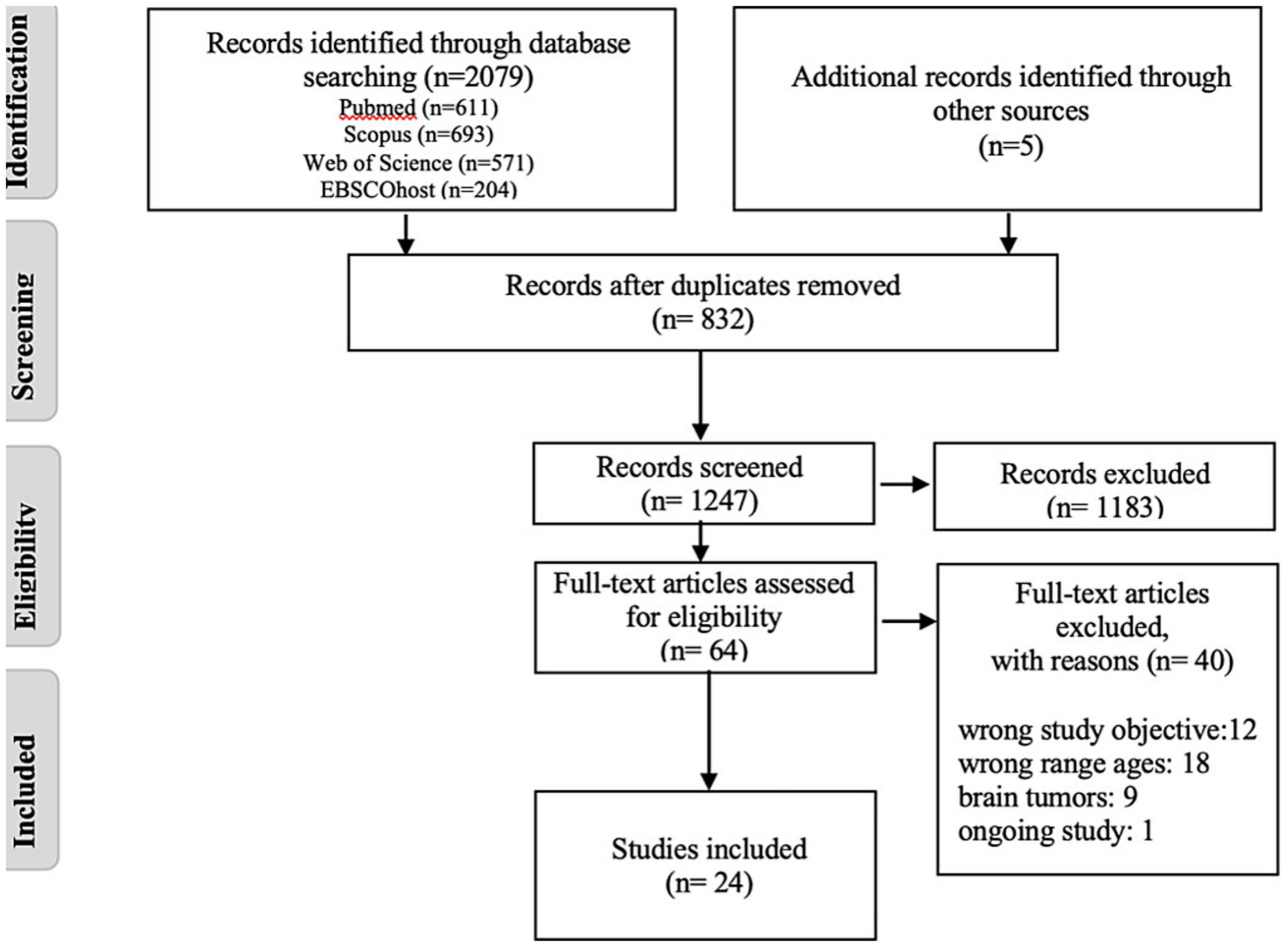
PRISMA-ScR Flow diagram for the scoping review process.

The search identified 2,079 records. After removing 832 duplicates, 1,247 titles and abstracts were screened, resulting in the exclusion of 1,183 records. The remaining 64 full-text articles were assessed for eligibility, and 40 were excluded. A total of 24 studies met the inclusion criteria and were included in the review. The selection process is summarized in the PRISMA flow diagram (Figure 1).

### Studies characteristics

The 24 selected studies were published from January 2014 to December 2024 and were conducted in 13 countries: Australia and New Zealand (*n* = 4), Canada (*n* = 4), United States (*n* = 3), Switzerland (*n* = 3), Sweden (*n* = 2), Germany (*n* = 2), France (*n* = 1), Netherlands (*n* = 1), UK (*n* = 1), Brazil (*n* = 1), Japan (*n* = 1), and South Korea (*n* = 1). Sample sizes ranged from *n* = 14 to *n* = 404, with a mean of *n* = 84 Participants’ ages ranged from 14 to 43 years old; they had finished treatment 1–12 years before, the majority 5 years before. All samples included male and female survivors of different types of cancer, mostly leukemias, lymphomas, and solid tumors. Regarding the study design, 14 were qualitative, using individual interviews (*n* = 12) or focus groups (*n* = 2); seven were quantitative cross-sectional, using questionnaire based survey (*n* = 2), and tests (*n* = 6); and three employed mixed methods using interviews (*n* = 3), and psychological tests (*n* = 2); and questionnaire-survey (*n* = 1). The quality scores for the qualitative studies ranged from 16 to 20; for quantitative studies, from 12 to 22; and for the mixed methods studies, from 27 to 31 (see Table 2).

**Table 2:** Study, objectives, design, measurement, outcomes, and quality assessment.

Publication	Study objectives	Design	Participants	Measurements	Main Outcomes	Quality Score *
Baudry et al. (2024), France	Explore the specific supportive care needs of childhood cancer survivors.	Qualitative	AYA-CCS 15–25Y 5 y post-diagnosis *N* = 15	Semi-structured interviews	AYAs need: 67% information on fertility issues and relapse anxiety 53.3% social assistance 20% guidance in the professional field 13.3% credit and insurance assistance.	17//20
Bradford et al. (2022), Australia	Understand the experience of late effects, quality of life, and modifiable factors.	Quantitative cross sectional study	CCS, ≥ 16 y (x̄ age 22 y) *N* = 30	FACT-G PROMIS	AYAs show lower QoL in physical and emotional well-being Symptoms: fatigue (53% in women), irregular menstrual periods (41.2%); memory problems (36.7%); concentration problems (33.3%); insomnia (33.3%), anxiety (30%).	21/22
Cherven et al. (2022), USA	Describe factors associated with fertility related worry.	Quantitative retrospective review	CCS, aged 18–26y ≥1y post-treatment *N* = 249	QAFRW	66.3% of CCSs reported concern about infertility. 75.5% of women reported worrying about infertility. Infertility concerns affect survivors’ lives and identity AYAs identify the need for psychological support.	20/22
Hendriks et al. (2020), Switzerland	Assess the unmet needs with regards to their long-term survivorship.	Qualitative	CCS, ≥ 18y ≥ 2y post-treatment (x̄ age, 31 y) N = 28	Semi-structured interviews	Unmet needs: Psychosocial support and information Centralised, specialised, interdisciplinary and individualised services. Resources for long-term follow-up.	18/20
Hendriks et al. (2022), Switzerland	Assess survivors’ wellbeing and care needs, and factors linked to unmet needs.	Mixed methods	CCS, ≥ 18y (69 CCS completed the survey; 28 were interviews) *N* = 69	*Qualitative*: Semi-structured interviews. *Quantitativ*e: SF-12, BSI-18; BCIA; HCNs	13% have poor physical HRQoL. 49% reported a poor mental HRQoL. 42% suffered from psychological disorders. AYAs felt various psychosocial, psychological, and informational needs were unmet	27/34
Howard et al. (2016), Canada	Identify how CCS manage medical and psychological challenges.	Qualitative	CCS, 22–43 y, ≥ 9y post-diagnostic *N* = 30	Interviews	How survivors manage health problems: Trying to forget about cancer Trusting the system in follow-up care Being proactive about health Struggling to find my way, felling lost and depressed Even when informed about possible late effects, CCS respond with shock, surprise, and anxiety when they occur.	16/20
Howard et al. (2018), Canada	Explore CCS and HCP views on healthcare system barriers to LTFU.	Qualitative	CCS, 19–45 y (*N* = 30 CCS *N* = 13 HCP) *N* = 30	Interviews	Obstacles to the transition of care: Difficult and abrupt transition from pediatric to adult health services, Inadequate GP experience in managing late effects, Late and insufficient communication of late effects.	17/20
Iwai et al. (2017), Japan	Examine CCS and family lifestyles, understanding of late effects, and problems across life stages.	Quantitative	CCS, 15–39 y, ≥ 5y post-tx (*N* = 30 CCS, *N* = 27parents) *N* = 30	Questionnaire survey, on mental-physical status and anxiety across life stages.	30% of AYAs responded that they were aware of the late effects. 20-30% of AYAs experienced mental and physical problems. Anxiety about passing the disease on to their children.	12/22
Jardim et al. (2021), Brazil	Understand the fertility-related concerns and uncertainties.	Qualitative	CCS, 18–24 y, > 5y post-treatment *N* = 24	Semi-structured interviews	Four themes: Fertility-related uncertainty has an emotional impact. Sharing the possible risk of infertility with partners has an impact (3) Need information about the possible loss of fertility	19/20
Lehmann et al. (2019), USA	Analyse how potential or confirmed infertility affects survivors’ romantic relationships.	Qualitative	CCS, 23–41 y, ≥12y post-diagnosis *N* = 30	Phone interviews	Impact of potential or confirmed infertility on romantic relationships Emotional reactions: worry, anguish, and guilt. Concerns about finding a partner and fear of rejection due to potential infertility.	18/20
Mayes et al. (2016), UK	Understand teenage and AYA survivors’ views on information about future health risks and prevention.	Qualitative	CCS, 14-33y ≥ 4y post-treatment *N* = 51	Semi-structured interview	37% want to receive more information about long-term effects. 59% see lifestyle counselling as more important for AYAs than for the general population. 55% believe oncologist/pediatrician should initiate health promotion. 88% view survivorship specialists are best for lifestyle counselling.	20/20
Newton et al. (2021), Canada	Describe the challenges of YACCS experience of living with an unknown fertility status.	Qualitative	CCS, 19–36 y, > 2 post-treatment *N* = 25	Interviews	YACCSs facing fertility doubts experienced difficulties: Ongoing psychological burden with anxiety and sadness. Negative impact on romantic and intimate relationships. YACCSs report limited and sometimes invalidated discussions with doctors.	20/34
Nilsson et al. (2020), Sweden	Explore how at-risk young adult survivors perceive their ability to have children	Qualitative	CCS, 17-27y ≥ 5y post-diagnosis *N* = 19	Interviews	Fertility concerns are postponed to feel normal. Having biological children signifies the body’s recovery after cancer. Concerns about heredity and passing cancer to children.	18/20
Nilsson et al. (2022), Sweden	Identify the needs and wishes for digitally mediated emotional peer support.	Qualitative	CCS, 19–33 y *N* = 14	Semi-structured interviews	AYAs identified needs: Processing long-term complications of cancer treatment; Processing psychosocial health; Desire to connect with peers and have a digital tool for survivors.	19/20
Otth et al. (2022), Switzerland	Evaluate CCS transition models by assessing knowledge, worries, self-management and LTFU.	Quantitative observational study: cross-sectional and longitudinal components.	CCS, > 16y, 5 y CCS *N* = 50	CWS; SMSS	AYAS are concerned: 44% possible late effects. 40% the possibility of not being able to have children. 42% agree that cancer is always on their mind. 25% recurrence. 24% and the development of a second neoplasm.	15/22
Patterson et al. (2021), Australia	Describe how psychosocial factors related to cancer treatment and infertility affect QoL.	Quantitative	CCS, 15–29 y *N* = 178	HRQOL	Higher QoL scores related to greater acceptance of the disease and older age. Lower infertility-related social concerns were directly and indirectly related to higher QoL scores. Parenthood desire linked to social concerns about infertility	21/22
Psihogios et al. (2019), USA	Understand AYA CCS experiences and preferences for survivorship care after healthcare transitions.	Qualitative cross-sectional study	CCS 15–29 y, (8 AYA, 6 YA not in LTFU) and 15 Parents *N* = 14	Eight focus groups (structured interview with 2 groups of AYA-CCS)	Priorities for AYAs: knowledge of late effects and transition of care; fear and uncertainty about health; autonomy and parental involvement considerations communication and coordination within the care team.	19/20
Signorelli et al. (2019), Australia and New Zealand	Explore survivors’ barriers to accessing and preferences for survivorship care.	Mixed methods	CCS, 187: CCS < 16 (parents); 251:16–25 y; 195. > 25 y *N* = 251	Questionnaires and optional interviews	Obstacles in the transition of care: Lack of knowledge about follow-up; Financial issues 54% of teens prefer children’s hospital; young adults prefer adult hospital. Young adults mainly requested psychologists and fertility specialists.	31/34
Van Erp et al. (2022), Netherlands	Assess CCS the support needs and psychosocial well-being with related socio-demographic, medical factors.	Quantitative cross-sectional	CCS, 18-30y, ≥ 5y post- diagnosis *N* = 151	PedsQL-YA; HADS; CIS-20R	76.2% for lifestyle and health risks after child cancer, 68.2% for fertility, 54.3% for insurance and mortgages, 53.6% for the socio-emotional consequences of cancer, 34.4% for relationships and sexuality, 29.8% for school and work. AYAS need information, counselling and peer contact.	22/22
Vetsch et al. (2020), Australia and New Zealand	To assess genetics-related information and service needs of survivors and parents.	Mixed methods	CCS, ≥ 16 y, 5y ≥ post-dx *Qualitative;* 52 CCS, 35 parents. *Quantitative:* 404 CCS, 218 parents *N* = 404	*Qualitative;* Telephone interviews *Quantitative:* Health EQ-5D-5L; questions on the following issues	84.6% of CCSs from families with a possible risk of genetic mutation 46.2% of AYAs with a possible risk of genetic mutation need information about the possibility of hereditary transmission 42% of both groups of survivors indicated that they believed it was ‘likely’/‘very likely’ that they had inherited a genetic mutation that caused the cancer.	30/34
Wang et al. (2015), Canada	Identify factors linked to cancer worry in AYA survivors and explore inappropriate worry.	Quantitative	CCS, 15–26 y, and completed cancer therapy *N* = 250.	CWS	54% of survivors were concerned about the risk of late effects. 31.5%of participants showed concern about the risk of reduced fertility and/or the development of a secondary malignant disease. Female were more concerned than male.	22/22
Winzig et al. (2024a), Germany	Exploring the impact of cancer and the burden on survival, the support needs of CCS in adolescence in LTFU care.	Qualitative	14–18 y Diagnosis of pediatric cancer Have finished treatment *N* = 18	Interviews	Types of concerns and needs of AYAs: physical consequences and cancer progression or recurrence difficulties in social interactions fertility and heredity issues lack of confidence discussing cancer with friends. overloading their sources of emotional support sharing with other CCS is a source of support.	20/20
Winzig et al. (2024b), Germany	Explore: pediatric cancer survivors’ attitudes towards follow-up care, and concerns about the transition process.	Qualitative	14–20 Y Diagnosis of pediatric cancer Have finished treatment *N* = 21	Interviews	AYAs concerns about transition care: Overwhelmed by living with the increased risk of long-term effects Not ready for transition, preference to continue receiving care in pediatric services Lack of involvement in the decision-making process, with parents being primarily involved.	20/20
Yi et al. (2016), Korea	Understand childhood cancer survivors’ worries.	Qualitative	CCS,19–39 y *N* = 28	Interviews	AYAs types of concerns: Romantic relationships and whether to disclose cancer history Concern about fertility and genetic risk Worry about cancer’s impact on future life and work Felt guilty for the family’s time spent caring for them Anxious about possibility cancer recurrence.	20/20

FACT-G, Functional Assessment of Cancer Therapy-General; PROMIS, Patient-Reported Outcomes Measurement Information System; QAFRW, n Questionnaire with an assessment of fertility-related worry; SF-12, Short Form Health Survey; BSI-18, Brief Symptom Inventory-18; BCIA, Brief Cancer Impact Assessment; HCNs, Health Care Needs survey; CWS, Questionnaire, Cancer Worries Survivors; SMSS, Self-management skills scale; HRQOL, Acceptance of illness, Infertility-related social concerns, and need for parenthood; PedsQL-YA, Pediatric Quality of Life -Young Adult; HADS, Hospital Anxiety and Depression Scale; CIS-20R, Checklist Individual Strength; EQ-5D-5L, Health Questionnaire. * Quality was assessed using Kmet’s checklist across quantitative, qualitative, and mixed-methods studies. The overall mean score was 89%; however, qualitative studies scoring below 75% were retained because they provided complementary thematic insights that contributed to conceptual saturation.

After selecting the articles, we mapped the studies’ results into two predefined main dimensions aligned with the review objectives: (1) concerns and difficulties, and (2) needs. Although “concerns,” “difficulties,” and “needs” were initially treated as distinct analytical concepts, drawing on APA definitions (APA, 2020), the literature rarely differentiated between “concerns” and “difficulties,” with most studies using these terms in overlapping ways to describe survivors’ psychosocial experiences. To ensure conceptual coherence and clarity in synthesis, we merged these into a single category of “concerns,” while maintaining “needs” as a distinct dimension referring to perceived gaps in support, resources, or services. Accordingly, the results were categorized into: (1) Concerns and (2) Needs.

**Figure 2:**
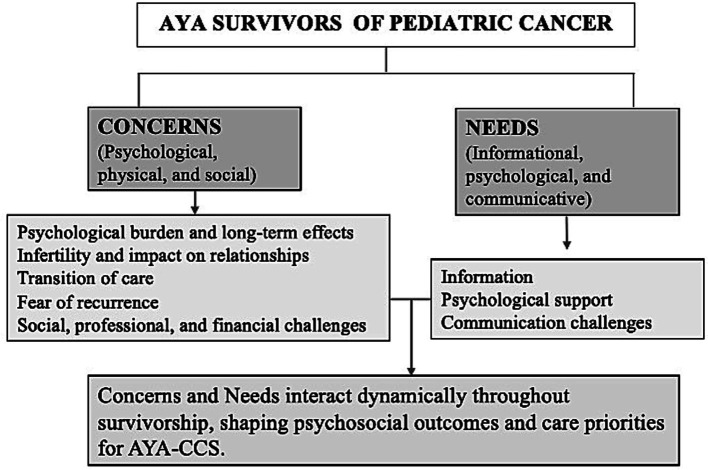
Conceptual map summarizing the main categories of concerns and needs among AYA-CCS.

The categorization of studies within these two overarching dimensions is summarized in Figure 2, which conceptually maps the main categories and illustrates their interrelations. A comprehensive list of studies supporting each subcategory is provided in Supplementary Table S1.

#### Concerns

This dimension encompasses the results of studies focused on the difficulties experienced by AYA-CCS during their adaptation to survival and their main concerns during this process. Concerns were assessed in 18 studies (Winzig et al., 2024a; Winzig et al., 2024b; Yi et al., 2016; Howard et al., 2016; Lehmann et al., 2019; Newton et al., 2021; Wang et al., 2015; Iwai et al., 2017; Bradford et al., 2022; Jardim et al., 2021; Nilsson et al., 2020; Cherven et al., 2022; Patterson et al., 2021; Baudry et al., 2024; Howard et al., 2018; Otth et al., 2022; Signorelli et al., 2019; Psihogios et al., 2019). Most (*n* = 11) were qualitative and used interviews and content analysis (Winzig et al., 2024a; Winzig et al., 2024b; Yi et al., 2016; Howard et al., 2016; Lehmann et al., 2019; Newton et al., 2021; Jardim et al., 2021; Nilsson et al., 2020; Baudry et al., 2024; Howard et al., 2018; Psihogios et al., 2019), while six were quantitative (Wang et al., 2015; Iwai et al., 2017; Bradford et al., 2022; Cherven et al., 2022; Patterson et al., 2021; Otth et al., 2022) and used sociodemographic questionnaires and tests, some of which used the Cancer Worries Scale (CWS) (Wang et al., 2015; Otth et al., 2022). One study combined one demographic questionnaire (with information about late effects) with interviews (Signorelli et al., 2019).

We identified concerns of young survivors in five categories: long-term effects/psychological burden; infertility/impact of fertility on relationships; transition of care; fear of recurrence; social, professional, and financial issues.

### Psychological burden and long-term effects

#### Psychological and emotional impact

Most studies describe how CCS patients deal with the long-term effects of treatment, particularly the psychological and emotional challenges that persist throughout survivorship (Winzig et al., 2024a; Winzig et al., 2024b; Yi et al., 2016; Howard et al., 2016; Lehmann et al., 2019; Newton et al., 2021; Wang et al., 2015; Iwai et al., 2017; Bradford et al., 2022). Across studies, survivors often report a sense of being overwhelmed by the ongoing risk of late effects (Winzig et al., 2024b; Howard et al., 2016; Wang et al., 2015; Iwai et al., 2017; Bradford et al., 2022), which contributes to a continuous psychological burden characterized by anxiety, distress, and guilt (Lehmann et al., 2019; Newton et al., 2021). Some participants internalize responsibility for the time and care invested by their families (Yi et al., 2016) Although these emotional responses are consistently reported, few studies differentiate by age, making it difficult to determine whether younger survivors experience or express psychological distress differently from older cohorts. This lack of age-specific analysis limits understanding of how developmental stage shapes coping strategies or perceptions of vulnerability.

#### Physical symptoms and wellbeing

Across studies, a substantial proportion of survivors report persistent physical and mental health problems, with estimates ranging from 20 to 30% (Iwai et al., 2017). AYA-CCS tend to show lower scores in HRQoL domains of physical and emotional well-being compared to the general population, with fatigue, menstrual irregularities, cognitive difficulties, joint pain, insomnia, and anxiety being among the most frequent symptoms (Bradford et al., 2022). Few studies differentiated by gender; some noted greater concern among women, but overall, differences in how men and women experience these concerns were not explored.

#### Expectations vs. reality of post-treatment effects

Some survivors reported not anticipating such persistent late effects and feeling unprepared to manage them, describing reactions of shock, surprise, anxiety, and worry (Howard et al., 2016). Even when informed about potential risks, this prior awareness did not always translate into adequate preparedness, as many continued to be startled when faced with late effects (Howard et al., 2016). Some studies reported that the information provided before and after treatment was not always sufficient to prepare survivors to deal with these experiences.

### Infertility and the impact of fertility issues on relationships

Infertility emerged as one of the most salient long-term concerns among young cancer survivors (Yi et al., 2016; Lehmann et al., 2019; Newton et al., 2021; Wang et al., 2015; Jardim et al., 2021; Nilsson et al., 2020; Cherven et al., 2022; Patterson et al., 2021; Baudry et al., 2024). In studies involving young adults, two-thirds (66.3%) reported fertility-related worries, particularly among women (Cherven et al., 2022), and these concerns were found to directly affect the quality of romantic and intimate relationships (Newton et al., 2021), while greater awareness of fertility risks and implications was observed among survivors who participated in multiple follow-up consultations (Cherven et al., 2022).

Emotional distress included feelings of fear, sadness, and uncertainty about reproductive capacity (Winzig et al., 2024a; Lehmann et al., 2019; Jardim et al., 2021). Limited understanding of post-treatment fertility and insufficient attention from healthcare professionals further contributed to this distress (Yi et al., 2016; Jardim et al., 2021). The ability to have biological children was described as a symbol of recovery and normalcy (Nilsson et al., 2020).

Lower social concern about infertility was associated with higher quality of life (Patterson et al., 2021). Fertility issues also generated anxiety about disclosing infertility or meeting partners’ expectations for parenthood (Lehmann et al., 2019; Newton et al., 2021; Jardim et al., 2021). Single survivors reported fear of rejection by potential partners due to infertility (Lehmann et al., 2019; Newton et al., 2021), and concern about passing on a genetic predisposition to cancer was also mentioned (Yi et al., 2016; Nilsson et al., 2020).

Most samples included both male and female survivors, but few studies differentiated fertility-related experiences by gender. Some indicated greater concern among women; however, overall, studies did not explore possible differences in how men and women experience these issues.

### Transition of care

Across all studies addressing this topic (Winzig et al., 2024a; Winzig et al., 2024b; Howard et al., 2016; Howard et al., 2018; Otth et al., 2022; Signorelli et al., 2019), the transition from pediatric to adult healthcare services was described by CCS as a difficult and significant challenge. The main difficulties reported related to establishing new relationships with healthcare professionals and to an abrupt transition, which led some survivors to feel abandoned and disoriented within the adult system, perceived as more complex (Howard et al., 2018), with a need for additional support in organizing follow-up appointments (Winzig et al., 2024a; Howard et al., 2018).

Overall, studies indicated that adolescent and young adult cancer survivors (AYA-CCS) received limited information about late effects and their management during the transition process (Howard et al., 2018).

Some survivors reported preferring to have their parents present during medical appointments (Otth et al., 2022), although they acknowledged that parental presence reduced their involvement in decision-making (Winzig et al., 2024b).

Regarding transition preferences, 54% of adolescents expressed a desire to remain under pediatric care, whereas young adults preferred adult services (Signorelli et al., 2019). These differences appear to reflect distinct needs associated with different developmental stages and the types of concerns that emerge during this period of transition, recognizing challenges in adapting to the transition of care (Howard et al., 2016).

### Fear of recurrence

Among AYA-CCS, other prevalent concerns were related to the fear of cancer recurrence (Winzig et al., 2024a; Wang et al., 2015; Baudry et al., 2024; Otth et al., 2022; Psihogios et al., 2019), the possibility of developing a second malignant neoplasm (Wang et al., 2015), and general uncertainty about future health (Psihogios et al., 2019; Mayes et al., 2016). These fears were frequently associated with persistent anxiety (Winzig et al., 2024a). In one study, 67% of survivors reported needing additional information and support related to recurrence anxiety (Baudry et al., 2024). Another study indicated that 13% of AYA-CCS expressed unrealistic concerns about developing a new cancer (Wang et al., 2015), while 42% reported that cancer was always on their mind and about one quarter worried about recurrence or the emergence of a second malignant disease (Otth et al., 2022). Overall, the studies indicate that uncertainty regarding health status and the risk of recurrence represents an ongoing source of psychological burden for many survivors.

### Social, professional, and financial issues

#### Social difficulties and stigmas

Other studies highlighted the social, professional, and financial difficulties experienced by survivors (Yi et al., 2016; Iwai et al., 2017; Baudry et al., 2024). One study reported concerns about limited employment opportunities associated with cancer-related stigma and reduced social skills, often linked to prolonged school absences or parental overprotection during illness (Yi et al., 2016). Overall, survivors expressed fear of restricted professional opportunities and social participation resulting from these factors (Yi et al., 2016). These difficulties appear to reflect broader challenges in social and professional reintegration after treatment, indicating persistent limitations in post-cancer adjustment.

#### Lack of social, academic and professional support

Another study of the specific needs of AYA-CCS reported that survivors required better coordination and multidisciplinary support across several areas, including social, administrative, financial, academic, and professional domains (Baudry et al., 2024). The study found that 53.3% of survivors required social assistance, 46.7% needed support with academic life, 40% need administrative assistance, and 20% sought guidance in the professional field (Baudry et al., 2024). Overall, these findings highlight the broad and interconnected nature of survivors’ post-treatment needs, underscoring the importance of integrated care approaches.

#### Financial concerns and access to resources

Concerns about financial issues are also present. Some participants expressed anxiety about obtaining or maintaining health insurance coverage, with most indicating worry about this matter (Iwai et al., 2017) Survivors also reported needing support and assistance in addressing difficulties related to access to credit and insurance (Baudry et al., 2024). Overall, these findings point to the persistence of financial insecurity as an additional burden in survivors’ post-treatment experience.

### Needs

Alongside the identification of difficulties and concerns, this study aimed to verify the AYA-CCS perspective about their needs regarding services and professional support, which they often view as unmet. A total of 10 studies were eligible to be analyzed (Winzig et al., 2024a; Cherven et al., 2022; Baudry et al., 2024; Psihogios et al., 2019; Mayes et al., 2016; Hendriks et al., 2022; Vetsch et al., 2020; Van Erp et al., 2022; Hendriks et al., 2020; Nilsson et al., 2022). This dimension includes results from six qualitative studies, which used semi-structured interviews (*n* = 5) (Winzig et al., 2024a; Baudry et al., 2024; Psihogios et al., 2019; Hendriks et al., 2020; Nilsson et al., 2022) and focus groups (*n* = 1) (Mayes et al., 2016), two quantitative studies that used sociodemographic questionnaires and tests (Cherven et al., 2022; Patterson et al., 2021; Baudry et al., 2024; Howard et al., 2018; Otth et al., 2022; Signorelli et al., 2019; Psihogios et al., 2019; Mayes et al., 2016; Hendriks et al., 2022; Vetsch et al., 2020; Van Erp et al., 2022), and two mixed methods studies with semi-structured interviews and sociodemographic questionnaires and tests (Psihogios et al., 2019; Mayes et al., 2016; Hendriks et al., 2022). We organized the needs of AYA-CCS in three categories: information, psychological support, and communication with their peers and health professionals.

### Information

One of the most frequently reported needs among survivors was access to personalized clinical and psychological information provided by specialists at the right time and in an appropriate format (Baudry et al., 2024; Mayes et al., 2016; Hendriks et al., 2022; Vetsch et al., 2020; Van Erp et al., 2022). Survivors reported requiring support and concrete information on lifestyle and health risks, physical and socio-emotional consequences of childhood cancer, fertility, and insurance-related issues (Van Erp et al., 2022). Several studies also showed the need for guidance on relationships and sexuality, educational and professional pathways, and future prospects (Baudry et al., 2024; Vetsch et al., 2020; Van Erp et al., 2022).

Regarding genetic information, survivors emphasized the importance of accurate and clearly communicated explanations about potential hereditary transmission, both among those with genetic risk and those without such risk (Vetsch et al., 2020). Other studies noted survivors’ interest in obtaining more information about the long-term treatment, effects and the importance they attached to lifestyle advice (Mayes et al., 2016). Research focusing on AYA-CCS highlighted the relevance of information on medical aspects of survivorship, including late effects, recurrence prevention, recommended examinations, and strategies for managing potential side effects (Baudry et al., 2024). However, several studies identified that the information needs remain largely unmet (Hendriks et al., 2022).

Overall, the studies converge in showing that AYA-CCS seek reliable, timely, and individualized information that addresses both medical and psychosocial dimensions of survivorship, revealing persistent gaps in communication and informational support.

### Psychological support

Five studies (Cherven et al., 2022; Baudry et al., 2024; Mayes et al., 2016; Hendriks et al., 2022; Hendriks et al., 2020) identified psychological support as an unmet need within healthcare services. AYA-CCS emphasized the importance of having access to multidisciplinary interventions that include psychological expertise (Signorelli et al., 2019). Across studies, survivors reported the need for psychological support to address various challenges of survivorship, including fertility-related distress (Cherven et al., 2022), reduced physical and mental quality of life, and psychological disorders (Baudry et al., 2024; Hendriks et al., 2022; Hendriks et al., 2020).

In addition, some studies indicated that AYA-CCS expressed the need for counselling focused on social, academic, and professional challenges, as well as on developing skills and lifestyle strategies to address the long-term effects of treatment (Mayes et al., 2016). Overall, the studies converge in showing that psychological support is consistently valued by survivors and described as a recurring need within survivorship care.

### Communication challenges

#### Communication with health professionals

Five studies have addressed the communication needs of survivors in their interactions health professionals (Winzig et al., 2024b; Baudry et al., 2024; Psihogios et al., 2019; Mayes et al., 2016; Nilsson et al., 2022). Survivors emphasized the importance of clear, open, and trusting communication with their care teams. Some survivors emphasized the importance of clear, open, and trusting communication with their care teams (Nilsson et al., 2022). Others reported difficulties explaining their medical history to new healthcare providers and obtaining support from former oncology teams (Nilsson et al., 2022). In one study, reluctance to transfer to adult services was linked to the strong communication and trust established with the pediatric care team (Mayes et al., 2016). Overall, these findings indicate that effective communication and coordination among healthcare professionals were consistently described as central to continuity and quality in survivorship care.

#### Communications with peers and family

In a qualitative study, AYA-CCS reported challenges in communicating with friends about their cancer experience and feelings of isolation in social interactions, while communication with parents was perceived as easier (Baudry et al., 2024). Sharing experiences with other survivors was described as a meaningful source of emotional support, helping them to normalize the experience of illness (Baudry et al., 2024). Some AYA-CCS expressed the wish to participate in peer-based initiatives or digital platforms that would enable interaction and mutual support in a safe environment (Nilsson et al., 2022).

In a study on the transition of care several AYA-CCS who preferred to be accompanied by their parents during medical appointments reported limited participation in discussions with the health care team, which reduced their involvement in decision-making processes (Winzig et al., 2024b). Together, these studies indicate that communication within families and peer networks is perceived as important for emotional well-being and for survivors’ engagement with care.

## Discussion

This review identified the difficulties, concerns, and needs of AYA-CCS. The findings contribute to updating and systematizing information to inform the development of psychological support programs aimed at aiding their adaptation. This understanding is crucial for implementing the recommendations of the Dublin Declaration (Childhood Cancer International, 2016) by CCI, which advocates for recognizing the complexity of survivorship and creating personalized support programs tailored to the specific needs of pediatric cancer survivors.

The analysis was structured around two *a priori* defined dimensions of the survivorship experience among AYA-CCS: psychological challenges, such as managing late effects, infertility, fear of recurrence, and social reintegration, and specific needs related to access to accurate information, psychological support, and the development of communication skills to enhance interpersonal relationships. To address these challenges, the study highlights the importance of personalized and flexible psychoeducational programs tailored to different age groups and individual needs. A significant methodological limitation observed across the reviewed studies is the lack of standardization in defining the AYA-CCS population, which may hinder data comparability and the formulation of precise clinical guidelines, underscoring the need for clear and consistent definitions.

### Addressing the concerns of AYA-CCS

The concerns expressed by AYA-CCS in this review reveal a persistent psychological burden linked to the late effects of cancer and their impact on mental health, daily functioning, and relationships (Winzig et al., 2024a; Winzig et al., 2024b; Yi et al., 2016; Howard et al., 2016; Lehmann et al., 2019; Newton et al., 2021; Wang et al., 2015; Iwai et al., 2017; Bradford et al., 2022; Jardim et al., 2021). This distress is compounded by symptoms such as fatigue, insomnia, and anxiety (Iwai et al., 2017; Bradford et al., 2022), and by awareness of long-term treatment consequences (Newton et al., 2021). Among these concerns, infertility stands out as a major issue affecting emotional well-being and normality, particularly among women, who report greater anxiety regarding family planning and parenthood (Lehmann et al., 2019; Newton et al., 2021; Jardim et al., 2021).

These findings align with the concept of continuous psychological burden described by López (2024) and supported by other authors (Warner et al., 2016; van Deuren et al., 2020). This burden encompasses fear of recurrence, academic and professional uncertainty, and financial insecurity (Winzig et al., 2024b; Yi et al., 2016; Wang et al., 2015; Iwai et al., 2017; Baudry et al., 2024; Psihogios et al., 2019). In line with broader survivorship literature, it also intersects with persistent symptoms such as fatigue and somatic worry, key factors influencing quality of life and fear of recurrence among childhood cancer survivors (Meeske et al., 2007; Levesque et al., 2023; Bower et al., 2014; Kelada et al., 2019; Cunningham et al., 2021).

Developmental tasks of adolescence and early adulthood, identity formation, autonomy, and intimate relationships (Maurice-Stam et al., 2022; Arnett, 2001), heighten AYA-CCS vulnerability, complicating the integration of the cancer experience into their life course. The transition from pediatric to adult care exposes tensions between dependence and autonomy; many survivors feel unprepared to navigate adult healthcare systems and remain reliant on parental involvement (Winzig et al., 2024b; Otth et al., 2022). As noted in the survivorship literature, this transition reflects not only organizational but also developmental challenges in autonomy and self-management, central to post-cancer adjustment (Wong et al., 2024).

Some survivors describe adaptive strategies such as “forgetting about cancer” or focusing on future goals, helping to restore continuity and control (Howard et al., 2016). Similar strategies in adolescents with chronic illness function as emotional regulation mechanisms supporting long-term adjustment (Compas et al., 2012; Mouw et al., 2017). Evidence from adult survivors shows that, although emotional and existential concerns persist, they often center on comorbidities, family responsibilities, and work reintegration (Bower et al., 2014). In contrast, AYA-CCS face these issues amid identity exploration, education, and pursuit of independence, amplifying the developmental impact of survivorship (Maurice-Stam et al., 2022; Arnett, 2001; Marchak et al., 2023).

This perspective underscores the need for survivorship care models integrating medical, psychological, and developmental dimensions, tailored to the specific challenges of this life stage.

### AYA-CCS needs: insights from the survivors

The studies included in this review identified a range of needs among AYA-CCS, revealing both addressed aspects and remaining gaps in survivorship care. A key concern is the demand for accurate, personalized information adapted to survivors’ developmental stage, life context, and disease trajectory. Survivors particularly value timely and relevant guidance on late treatment effects, fertility preservation, genetic counselling, and health promotion, as information perceived as inadequate or poorly timed tends to be disregarded, weakening engagement in care (Baudry et al., 2024; Mayes et al., 2016; Hendriks et al., 2022; Vetsch et al., 2020; Van Erp et al., 2022).

Previous survivorship research highlights the central role of information and communication for continuity of care and transition to adult services (Mouw et al., 2017; Marchak et al., 2023; Schmidt et al., 2020; Sadak et al., 2021). However, many survivors still report surprise and distress when facing late effects, suggesting persistent gaps in the quality and consistency of communication (Howard et al., 2016). The frequent expression of surprise indicates that existing information on long-term adverse outcomes may be insufficient or poorly framed, suggesting that communication approaches may not adequately address survivors’ developmental or emotional needs. This discrepancy is particularly relevant, as lower knowledge levels have been associated with greater psychological distress in broader survivorship literature (Marchak et al., 2023), underscoring the importance of clinically grounded information and psychological support to foster effective coping.

Difficulties in communication with healthcare providers, family, peers, and partners are also frequently reported (Winzig et al., 2024b; Jardim et al., 2021; Baudry et al., 2024; Nilsson et al., 2022). Survivors emphasize the importance of open, trusting communication throughout follow-up, which supports emotional adjustment and self-efficacy (Howard et al., 2016; Hendriks et al., 2022). These findings reinforce the value of integrated care models that combine information, psychological support, and communication skill development.

In line with recent survivorship literature, studies with adult cancer survivors also emphasize holistic, multidisciplinary approaches that integrate educational, psychological, and physical components to enhance adaptation (Walsh et al., 2024; O’Connor, 2019). Within this framework, psychoeducational programs emerge as a promising means of supporting AYA-CCS in managing the multiple demands of survivorship. The results of our review confirm the relevance of such interventions, which equip survivors to address the complex emotional, social, and practical challenges of post-cancer life. Consistent with evidence from other chronic conditions, psychoeducational interventions have proven effective in improving coping and psychosocial adjustment (Compas et al., 2012; Phiri et al., 2023; Cortesi, 2020).

Considering the developmental tasks of adolescence and young adulthood, such as identity consolidation, autonomy, and relational development (Maurice-Stam et al., 2022; Arnett, 2001), integrating these dimensions into survivorship programs is essential. Although some concerns overlap with those of adult cancer survivors, the needs of AYA-CCS are qualitatively distinct, shaped by ongoing developmental transitions and the pursuit of identity, independence, and future goals. Approaches addressing these interconnected domains can enhance adaptive functioning and quality of life, ensuring that interventions respond not only to medical and psychological consequences but also to the developmental context of this life stage.

### Guidelines for developing intervention programs addressing the concerns and needs of AYA-CCS

Our findings underscore the need for multifaceted interventions addressing the diverse challenges faced by AYA-CCS. Given the substantial psychological burden during survivorship, programmatic themes should comprehensively include coping with long-term effects, emotional and physical health, communication issues, and preparation for the future.

Psychoeducational programs for AYA-CCS should be tailored to their specific needs, with flexible structures that allow individualization. A modular approach is particularly suitable, enabling the selection of relevant content based on age, developmental stage, and personal challenges (Cortesi, 2020). Evidence from recent reviews supports the use of both face-to-face and technology-based interventions, as well as adaptable formats that integrate psychoeducation, coping skills, and health promotion (Arpaci and Altay, 2024). Practical frameworks have been proposed to guide such approaches. For instance, Arpaci et al. (2022) developed a technology-based psychoeducational program for adolescent survivors aged 12–18 years, organized into interactive modules on self-knowledge, communication, coping, problem-solving, and health promotion. This example illustrates how modular, developmentally tailored programs can flexibly address diverse psychosocial needs across ages and individual challenges (Arpaci et al., 2023).

To enhance developmental relevance, interventions could be structured by age bands (e.g., 15–18, 19–24, and 25–39 years), reflecting predominant concerns at each stage. Issues of autonomy and communication may be central for younger groups, including modules directed to parents, whereas for older survivors, topics such as fertility, family planning, and career management may become more salient.

### Methodological inconsistencies in the definition of AYA-CCS: implications for research and survivorship care

A persistent methodological limitation in pediatric cancer survivorship research is the lack of standardized criteria for defining adolescent and young adult childhood cancer survivors (AYA-CCS). The studies reviewed show considerable variation in age ranges and diagnostic timing. Some define AYA-CCS as individuals diagnosed in childhood who have since transitioned into adolescence or young adulthood, while others adopt the U.S. National Cancer Institute (NCI) classification of AYA as those diagnosed between ages 15 and 39, further subdivided into 15–18, 19–24, and 25–39 years (Janssen et al., 2021). Yet, this framework is inconsistently applied, with some studies using broader or atypical age spans (e.g., 17–43y, Howard et al., 2016; 19–33y, Newton et al., 2021) or thresholds beginning at 16 or 18 years.

Such inconsistencies hinder cross-study comparability and limit the development of evidence-based interventions. Grouping adolescents and young adults without regard to developmental stage risks obscuring critical differences in psychosocial needs. Only three studies (Cherven et al., 2022; Mayes et al., 2016; Nilsson et al., 2022) in our review stratified findings by developmental subgroups, reflecting a wider trend of undifferentiated analysis.

This issue was identified over a decade ago (Geiger and Castellino, 2011), yet remains unresolved, compounded by variation in age classifications across countries. A further consequence is the frequent exclusion of survivors diagnosed before age 15 from AYA-focused studies, despite their later transition into adolescence or adulthood and their exposure to similar survivorship challenges.

A unified classification system for AYA-CCS, grounded in both age and developmental stage, is urgently needed to improve consistency, enhance data interpretation, and inform the design of developmentally appropriate psychosocial care.

## Limitations

This scoping review has several limitations.

First, only primary research studies were included, as the aim was to analyze data directly reported by AYA-CCS. This approach ensured proximity to survivors’ self-reported experiences but may have excluded valuable insights from reviews or conceptual papers addressing broader contextual or theoretical dimensions.

Second, we included only studies published in English. This restriction may have introduced a language and cultural bias, as it can lead to the underrepresentation of non-Western perspectives and culturally diverse experiences of AYA-CCS. Consequently, the findings of this review may primarily reflect Western contexts and healthcare systems, which could limit the transferability of these conclusions to settings with different cultural values, health beliefs, or survivorship frameworks.

Third, relevant information may have been missed by excluding studies that did not clearly distinguish the perspectives of young survivors from those of older adult survivors, family members, or professionals. This may have limited the breadth of perspectives captured and could influence the comprehensiveness of the mapped evidence.

Finally, the review protocol was not prospectively registered, as PROSPERO currently does not accept scoping review registrations. While this may reduce external transparency, all methodological procedures were defined *a priori*, discussed collaboratively among the three authors, and cross-checked to ensure consistency, thus providing a form of methodological triangulation that strengthens internal rigor. The review was also conducted in strict adherence to the PRISMA-ScR framework, with systematic and predefined procedures to minimize potential bias.

Collectively, these limitations may affect the generalizability of the findings, particularly across different cultural and healthcare contexts. Nevertheless, they do not undermine the main contribution of this review, which offers a comprehensive synthesis of AYA-CCS self-reported difficulties, concerns, and needs, providing a robust foundation for future research and intervention development.

## Conclusion

This study offers innovative contributions to the research on adolescent and young adult survivors of pediatric cancer (AYA-CCS) by comprehensively highlighting their specific needs and concerns, while proposing concrete pathways for the development of more effective interventions. It emphasizes the importance of creating personalized, modular, and flexible psychoeducational programs that account for different age groups and individual demands within this population.

Furthermore, this review identifies a recurrent methodological limitation: the lack of standardization in defining the AYA-CCS population, which undermines data comparability and the formulation of precise clinical guidelines. By underscoring the need for uniform methodological criteria and the active involvement of survivors in the development of care strategies, this work opens new avenues for a more sensitive, accurate, and effective approach in this field.

## Footnotes

1
rayyan.qcri.org
