# Supplementary material for: Identifying concerns and needs in AYA survivors of pediatric cancer: a scoping review
Source: Front Psychol. 2025 Dec 4;16:1669872. doi: 10.3389/fpsyg.2025.1669872 (PMC12713121; doi:10.3389/fpsyg.2025.1669872)
Supplement: Supplementary file 2 [file Supplementary_file_2.docx]

**Quality assessment of quantitative, mixed-methods studies, and qualitative**

| Quality assessment of quantitative studies | Bradford et al.,  2022 | Iwai  et al., 2016  J | Cherven et al.,  2022 | Otth  et al.,  2022 | Patterson et al.,  2021 | Van Erp  et al.,  2022 | Wang et al.,  2015 |
| --- | --- | --- | --- | --- | --- | --- | --- |
| 1. Question / objective sufficiently described? | 2 | 0 | 2 | 2 | 2 | 2 | 2 |
| 1. Study design evident and appropriate? | 2 | 1 | 1 | 1 | 2 | 2 | 2 |
| 1. Method of subject/comparison group selection or source of information/input variables described and appropriate? | 2 | 2 | 2 | 2 | 2 | 2 | 2 |
| 1. Subject (and comparison group, if applicable) characteristics sufficiently described? | 2 | 2 | 2 | 2 | 2 | 2 | 2 |
| 1. If interventional and random allocation was possible, was it described? | NA | NA | NA | NA | NA | NA | NA |
| 1. If interventional and blinding of investigators was possible,was it reported? | NA | NA | NA | NA | NA | NA | NA |
| 1. If interventional and blinding of subjects was possible,was it reported? | NA | NA | NA | NA | NA | NA | NA |
| 1. Outcome and (if applicable) exposure measure(s) well defined and robust to measurement / misclassifi cation bias? Means of assessment reported? | 2 | 1 | 2 | 2 | 2 | 2 | 2 |
| 1. Sample size appropriate? | 1 | 1 | 2 | 2 | 2 | 2 | 2 |
| 1. Analytic methods described/justified and appropriate? | 2 | 1 | 2 | 1 | 2 | 2 | 2 |
| 1. Some estimate of variance is reported for the main results? | 2 | 0 | 2 | 0 | 1 | 2 | 2 |
| 1. Controlled for confounding? | 2 | 0 | 2 | 0 | 2 | 2 | 2 |
| 1. Results reported in sufficient detail? | 2 | 1 | 2 | 1 | 2 | 2 | 2 |
| 1. Conclusions supported by the results? | 2 | 1 | 2 | 2 | 2 | 2 | 2 |
| **Total** | 21/22 | 12/22 | 20/22 | 15/22 | 21/22 | 22/22 | 22/22 |

| Quality assessment of mixed-methods studies | Hendriks et al.,  2022 | Signorelli et al.,  2019 | Vetsch et al.,  2020 |
| --- | --- | --- | --- |
| 1. Question / objective sufficiently described? | 2 | 2 | 2 |
| 1. Study design evident and appropriate? | 2 | 2 | 2 |
| 1. Context for the study clear? | 2 | 2 | 2 |
| 1. Connection to a theoretical framework / wider body of knowledge? | 1 | 1 | 1 |
| 1. Sampling strategy described, relevant and justified? | 2 | 2 | 2 |
| 1. Data collection methods clearly described and systematic? | 2 | 2 | 2 |
| 1. Data analysis clearly described and systematic? | 2 | 2 | 1 |
| 1. Use of verification procedure(s) to establish credibility? | 2 | 2 | 1 |
| 1. Conclusions supported by the results? | 2 | 2 | 2 |
| 1. Reflexivity of the account? | 1 | 1 | 1 |
| 1. Method of subject/comparison group selection or source of information/input variables described and appropriate? | 2 | 2 | 2 |
| 1. Outcome and (if applicable) exposure measure(s) well defined and robust to measurement / misclassifi cation bias? Means of assessment reported? | 2 | 2 | 2 |
| 1. Sample size appropriate? | 2 | 2 | 2 |
| 1. Analytic methods described/justified and appropriate? | 1 | 2 | 2 |
| 1. Some estimate of variance is reported for the main results? | 1 | 1 | 2 |
| 1. Controlled for confounding? | 0 | 2 | 2 |
| 1. Results reported in sufficient detail? | 1 | 2 | 2 |
| **Total** | 27/34 | 31/34 | 30/34 |

| Quality assessment of qualitative studies | Baudry  et al,  2024 | Hendriks et al.,  2021 | Howard et al.,  2016 | Howard et al.,  2018 | Jardim et al.,  2021 | Lehmann et al.,  2019 | Mayes et al.,  2016 | Nilsson et al.,  2020 | Newton et al.,  2021 | Nilsson et al.,  2022 | Psihogios et al.,  2019 | Yi  et al, 2016 | Winzig  et al,  2023 | Winzig  et al,  2024 |
| --- | --- | --- | --- | --- | --- | --- | --- | --- | --- | --- | --- | --- | --- | --- |
| 1. Question / objective sufficiently described? | 1 | 2 | 2 | 2 | 2 | 1 | 2 | 2 | 2 | 2 | 2 | 2 | 2 | 2 |
| 1. Study design evident and appropriate? | 1 | 2 | 2 | 2 | 2 | 2 | 2 | 2 | 2 | 2 | 2 | 2 | 2 | 2 |
| 1. Context for the study clear? | 2 | 2 | 2 | 2 | 2 | 2 | 2 | 1 | 2 | 2 | 2 | 2 | 2 | 2 |
| 1. Connection to a theoretical framework / wider body of knowledge? | 2 | 2 | 2 | 2 | 1 | 1 | 2 | 2 | 2 | 1 | 1 | 2 | 2 | 2 |
| 1. Sampling strategy described, relevant and justified? | 2 | 2 | 2 | 2 | 2 | 2 | 2 | 2 | 2 | 2 | 2 | 2 | 2 | 2 |
| 1. Data collection methods clearly described and systematic? | 2 | 1 | 2 | 2 | 2 | 2 | 2 | 2 | 2 | 2 | 2 | 2 | 2 | 2 |
| 1. Data analysis clearly described and systematic? | 2 | 1 | 2 | 1 | 2 | 2 | 2 | 2 | 2 | 2 | 2 | 2 | 2 | 2 |
| 1. Use of verification procedure(s) to establish credibility? | 1 | 2 | 0 | 0 | 2 | 2 | 2 | 2 | 2 | 1 | 2 | 2 | 2 | 2 |
| 1. Conclusions supported by the results? | 2 | 2 | 2 | 2 | 2 | 2 | 2 | 2 | 2 | 2 | 2 | 2 | 2 | 2 |
| 1. Reflexivity of the account? | 2 | 2 | 1 | 2 | 1 | 2 | 2 | 1 | 1 | 2 | 2 | 2 | 2 | 2 |
| Total | 17/20 | 18/20 | 16/20 | 17/20 | 19/20 | 18/20 | 20/20 | 18/20 | 19/20 | 18/20 | 19/20 | 20/20 | 20/20 | 20/20 |
