# Supplementary material for: Identifying concerns and needs in AYA survivors of pediatric cancer: a scoping review
Source: Front Psychol. 2025 Dec 4;16:1669872. doi: 10.3389/fpsyg.2025.1669872 (PMC12713121; doi:10.3389/fpsyg.2025.1669872)
Supplement: Supplementary file 3 [file Table_1.docx]

**Supplementary Table S1:** Categorization of concerns and needs

| **Category** | **Subcategories** | **Studies** |
| --- | --- | --- |
| **Concerns** | Concerns about long-term effects | 27, 28, 29, 30, 31, 32, 33, 34, 35 |
|  | Infertility/ impact of fertility issues on relationships | 29, 31, 32, 33, 36, 37, 38, 39,40 |
|  | Transition of care | 27,28,30,41, 42,43 |
|  | Fear of recurrence | 27,33,40,42,44 |
|  | Social, professional, and financial issues | 29,34,40 |
| **Needs** | Information | 40, 45, 46,47, 48 |
|  | Psychological support | 38, 40, 45, 46,49 |
|  | Communication challenges | 28,40,44,45,50 |
